# Microbial alkane production for jet fuel industry: motivation, state of the art and perspectives

**DOI:** 10.1111/1751-7915.12423

**Published:** 2016-10-10

**Authors:** Lorena Jiménez‐Díaz, Antonio Caballero, Natalia Pérez‐Hernández, Ana Segura

**Affiliations:** ^1^ Abengoa ResearchCampus Palmas Altas, C/Energía Solar41014SevillaSpain; ^2^ Estación Experimental del Zaidín‐CSICC/Profesor Albareda s/n18008GranadaSpain

## Abstract

Bio‐jet fuel has attracted a lot of interest in recent years and has become a focus for aircraft and engine manufacturers, oil companies, governments and researchers. Given the global concern about environmental issues and the instability of oil market, bio‐jet fuel has been identified as a promising way to reduce the greenhouse gas emissions from the aviation industry, while also promoting energy security. Although a number of bio‐jet fuel sources have been approved for manufacture, their commercialization and entry into the market is still a far way away. In this review, we provide an overview of the drivers for intensified research into bio‐jet fuel technologies, the type of chemical compounds found in bio‐jet fuel preparations and the current state of related pre‐commercial technologies. The biosynthesis of hydrocarbons is one of the most promising approaches for bio‐jet fuel production, and thus we provide a detailed analysis of recent advances in the microbial biosynthesis of hydrocarbons (with a focus on alkanes). Finally, we explore the latest developments and their implications for the future of research into bio‐jet fuel technologies.

## Motivation

### Aviation industry and environment

Experts of the World Economic Forum have indicated that climate change is one of the main threats of economic growth ( https://www.weforum.org/reports/the-global-risks-report-2016). In May 2016, NASA revealed that CO_2_ levels—which are directly related to climate change—had reached a historic high of 404.36 ppm ( http://climate.nasa.gov/vital-signs/carbon-dioxide/). The combustion of fossil fuels is the second largest source of CO_2_ emissions, representing 31% of all US CO_2_ emissions and 25% of all EU emissions. Of these global emissions, around 2–3% are due to air transportation ( http://www.eutransportghg2050.eu/cms/the-contribution-of-transport-to-ghg-emissions/). While most greenhouse gas (GHG) emissions from other sectors are generally falling, those from transport increased by 36% between 1990 and 2007. This is in part because the air transportation market is expanding at the average growth rate of 5% of passenger volumes yearly ( http://www.iata.org/publications/economics/, Moriarty and Honnery, 2008).

Fuel constitutes up to 28% of an airline's operating cost (Hong *et al*., 2013; Gegg *et al*., 2014), and because of oil price volatility, airline operators find very difficult to plan and budget for long‐term operating expenses. The necessity to reduce risk in the fuel price volatility is a fundamental driver for the aviation industry to develop bio‐jet fuels from renewable sources.

In March 2010, the European Commission, as part of its *Europe 2020* strategy (European Commission, 2010), highlighted the need to decarbonise transport, and in doing so linked this objective with the wider sustainable growth agenda. Similar guidelines are on the agenda of most of the world countries. The International Air Transport Association (IATA) established a roadmap for the reduction of CO_2_ emissions that included improvement of fuel efficiency by 1.5% annually to achieve carbon neutral growth by 2020 and cut by 50% the emissions in 2050 ( www.iata.org). To achieve this reduction in carbon footprint, improvement in new technologies, operation proficiency, efficient infrastructure and market‐based measures were required—aims that demanded the involvement of oil companies, and aircraft and engine manufacturers. Each of the main stakeholders had different motivations to invest in bio‐jet fuel research. For oil & gas companies, research did not represent a high cost and would allow them to be ready to quickly dominate the market when bio‐jet fuels are commercialized; the airline industry's main interest was in reducing their dependability on a single source of fuel and in helping the industry to meet their 2050 IATA CO_2_ emissions target; for aircraft manufacturers, the main goals were to reduce the environmental impact of air transportation and to improve jet‐fuel quality and prevent turbine failures. All these interests converge and have resulted in intensified interest in aviation research, including research into bio‐jet fuel. Although many engineering research efforts are currently focused on improving engines to achieve better fuel efficiencies, aircraft manufacturers are playing an active role and are engaged with American Society for Testing and Materials (ASTM) to approve bio‐jet fuel.

Given the global concern for environmental issues, fuel producers have committed to carry out not only conventional techno‐economic analysis of specific bio‐jet fuel production processes, but also analysis of their impacts on global GHG emissions. This balance is called life cycle assessment (LCA) and is normally performed from the field, where the feedstock is harvested, to its utilization as aircraft fuel (Stratton *et al*., 2010). Factors such as the cumulative energy demand for each bio‐fuel, cultivation methods, chemicals used and transportation are taken into account. A LCA analysis of the hydroprocessed renewable jet (HRJ) emissions from camelina crops demonstrated that the largest contribution came from feedstock production, biofuel production, feedstock chemicals and feedstock transport in descending order. CO_2_ produced during the combustion of the fuel was considered carbon neutral as it comes from CO_2_ fixed by the plant from the atmosphere (Shonnard *et al*., 2010). LCA from different renewable processes demonstrated that CO_2_ emissions could be reduced by up to 80% depending on the production method and the type of fuel being considered (Koers *et al*., 2009; IATA, 2010). This means that, by blending conventional fuel with 1% of bio‐jet fuel, the aviation industry could reduce overall emissions by up to 0.8% (Hong *et al*., 2013). In addition, bio‐jet fuels produce lower emissions of other important GHGs, such as SO_x_, NO_x_ and other particulate matter, which represents an added advantage versus conventional jet fuels. Taken together, these drivers demonstrate that the research and development of new and more efficient bio‐jet fuel synthesis methods should be made a major priority.

Aviation fuels must meet a number of stringent specifications—the most significant of which is achieving the high energy density required to maximize aircraft range and to ensure low freezing‐points, which prevent the formation of crystalline wax particles during high‐altitude flight (Hemighaus *et al*., 2007; Blakey *et al*., 2011; Chuck and Donnelly, 2014). The main types of jet fuel used today are kerosene‐based (Jet A and Jet A‐1) and gasoline‐based (Jet B). Jet B includes all light hydrocarbon oils for use in aviation, which distill between 100°C and 250°C (International Energy Agency http://www.iea.org/). Kerosene type jet fuels (Jet A and Jet A1) are mixtures of hydrocarbons, which are mainly alkanes, which include linear hydrocarbons also known as paraffins, branched alkanes, cycloalkanes, olefins and aromatic hydrocarbons. The presence of branched alkanes helps to decrease boiling and freezing points. Aliphatic, branched and cyclo‐alkanes typically represent up to 70% to 80% (by volume) of the kerosene type of jet fuel (Blakey *et al*., 2011; Kallio *et al*., 2014a). Hence, alkanes have been targeted as the critical product to develop through biological synthesis, and most of the recent scientific literature on bio‐jet fuel synthesis identifies alkanes as the desired product.

Initially, there were two methods developed for the renewable production of alkanes. One of them was based mainly on the utilization of oleaginous crops to obtain triglycerides and fatty acids that were later chemically converted to alkanes. A second method was based on biomass pyrolysis and syngas production followed by successive chemical steps for alkane synthesis (Hari *et al*., 2015; see below). Although these processes reached pre‐commercial stages, they have several technological, economic and socio‐political drawbacks (discussed below) that must be resolved. Remarkably, microorganisms are natural producers of medium‐and long‐chain fatty acids and some of them are able to convert these fatty acids into alkanes and/or alkenes. The biotechnology boom and the popularity of industrial biological processes have created an environment where research into the microbial production of alkanes has taken off.

### Pre‐commercial processes for bio‐jet fuel production

Currently, there exist only two alternative aviation fuels approved by the ASTM: Bio Derived Synthetic Paraffinic Kerosene (Bio‐SPK) that is produced from oil through hydroprocessed renewable jet technology (HRJ) and Fischer‐Tropsch Synthesis Paraffin Kerosene (FT‐SPK), generated from gas [gas‐to‐jet GTJ)]. The different existing technologies to produce renewable jet fuels have been recently reviewed (Hari *et al*., 2015; Wang and Tao, 2016) and are briefly described in Table 1.

**Table 1:** Classification of bio‐jet fuel conversion technologies (adapted from in Wang and Tao, 2016). Conversion pathways are classified on the basis of the starting material (alcohol, oil, gas and sugars). The different processes used to convert the starting materials into alkanes are depicted in fundamentals and the different steps in the process briefly described in the last column. HRJ and FT‐SPK are the renewable technologies approved by the ASTM. The pro bio‐jet fuel from those processes are labelled as Bio‐SPK and FT‐SPK respectively

Conversion pathways	Fundamentals	Description
ATJ (or alcohol isomerization)	Upgrading short chain alcohols and long‐chain fatty alcohols to fuel	Alcohol dehydration, oligomerization, hydrogenation
Oil‐to‐jet	(a) HRJ: employ triglyceride‐based feed‐stocks to produce FFAs by propane cleavage	Hydrogenation, deoxygenation, hydro‐isomerization and hydrocracking
	(b) CH or hydrothermal liquefaction: employ triglyceride‐based feed‐stocks to produce FFAs by thermal hydrolysis	Cracking, hydrolysis, decarbonylation, isomerization and cyclization
	(c) Hydro‐treated depolymerized cellulosic jet or fast pyrolysis with upgrading to jet fuel	Oils from pyrolysis undergo hydro‐treatment and fractionation
Gas‐to‐jet	(a) FisherTropsch Biomass To Liquid (FT‐SPK)	Biomass is dried and converted to syngas that is polished, activated, hydro‐processed and finally converted to liquid hydrocarbons through F‐T synthesis
	(b) Gas fermentation process	Biomass is converted to syngas and fermented to ethanol or butanol that is upgraded via ATJ
Sugar‐to‐jet	(a) Catalytic upgrading of sugars or sugar intermediates	Sugars are separated from biomass and upgrading through aqueous phase reforming
	(b) Fermentation of sugars to hydrocarbons (direct sugar to hydrocarbons)	Sugars from biomass are fermented to hydrocarbons

HRJ, hydroprocessed renewable jet; FT‐SPK, Fischer‐Tropsch Synthesis Paraffin Kerosene; ASTM, American Society for Testing and Materials; Bio‐SPK, Bio Derived Synthetic Paraffinic Kerosene; ATJ, alcohol‐to‐jet; FFA, free fatty acid; CH, catalytic hydrothermolysis.

Bio‐SPK produced by HRJ technology is synthesized from oil and is therefore within the group of technologies known as oil‐to‐jet (OTJ). Typically, Bio‐SPK is produced through a process that involves the transesterification of triglycerides and fatty acids from vegetable, animal or waste oils, followed by hydrogenation, hydrocracking and hydroprocessing to generate alkanes of desired length (C_8_ to C_16_), saturation level and branching (Kallio *et al*., 2014a).

In addition to the hydro‐treated esters and fatty acids (HEFA) also known as HRJ and mentioned above, OTJ synthesis schemes include catalytic thermolysis (CH) or hydrothermal liquefaction; and the hydro‐treated depolymerized cellulosic jet process (Table 1).

Hydroprocessed renewable jet/HEFA utilizes triglycerides from different raw materials (fats and oils from vegetables, animals or waste oil). Triglycerides make up the structure of all vegetable oils and fats found in nature and they are composed of a glycerol molecule linked to three esterified fatty acids. These fatty acids can be saturated, monounsaturated or polyunsaturated and have different chain lengths. Depending on the raw material used in bio‐jet fuel processes, the resulting fatty acid content varies significantly. For example, Jatropha contains the following proportions of fatty acids: 16% palmitic (C16:0); 1% palmitoleic (C16:1), 7% stearic (C18:0); 41% oleic (C18:1) 34.7% linoleic (C18:2). Palm contains significantly more palmitic acid (41%) and less linoleic acid (7.9%) therefore, higher proportion of saturated fatty acids. Because of the different degrees of unsaturation, one hydrogenation step is required to completely saturate the double bonds and the conditions of this hydrogenation will differ depending on the raw material (Sotelo‐Boyás *et al*., 2012). Catalytic hydrogenation converts triglycerides into saturated fatty acids and glycerides; successive cracking will produce glycerol that is turned into propane by adding H_2_ and three molecules of fatty acid (Mohammad *et al*., 2013). An alternative route is thermal hydrolysis; triglycerides are thermally hydrolysed to form saturated and unsaturated free fatty acids (FFA) and glycerol. FFA are then thermo‐catalytically decarboxylated to alkanes; glycerol can be purified and sold for pharmaceutical, technical and personal care products, increasing the value of the process (Wang *et al*., 2012).

The fuel obtained directly by hydrodeoxygenation is mainly composed of C_17_ and C_18_ *n*‐paraffins that have a high cetane number but poor cold flow properties; to meet jet fuel specifications, it is necessary to hydrocrack and hydroisomerize the paraffins. In this process, the oxygen is removed as water, the deoxygenated product is separated by distillation and the heavier molecules are cracked to produce paraffins that satisfy jet fuel specifications (C_9_ to C_16_). Hydroisomerization is mainly used to produce branched alkanes (Sotelo‐Boyás *et al*., 2012).

Most of this process has to be carried out at high temperatures and pressures; and requires large amounts of hydrogen, and bifunctional chemical catalysts containing metallic sites for hydrogenation/dehydrogenation and acid sites for isomerization (Sotelo‐Boyás *et al*., 2012; Wang and Tao, 2016). The selection of catalyst and temperature are important factors in order to obtain the best product.

From an industrial point of view, the production of green fuels from triglycerides requires standard equipment (e.g., catalysts, reactor type, separation equipment and others) that has been in use in petroleum refineries for the past 60 years. Hydrogenation of vegetable oils is a technology that is also used frequently in food industry. Hence, most of the main R&D efforts have been focused on the optimization of unique production and operating conditions.

Hydroprocessed renewable jet has been already tested for military flights (Rye *et al*., 2010), and approved by the ASTM. Thus, it is expected to be a viable commercial option soon. The production of Bio‐SPK (HRJ) is relatively high efficient, but the cost to produce it is subjected to the expensive hydrogenation step, which consumes large amounts of hydrogen. The final product has lower sulfur content and potentially lower GHG emissions than conventional jet fuels; however, its lower heating value and higher freezing point, together with the presence of contaminants (e.g., metals) carried forward from the raw material represent serious challenges that must still be addressed (Bradshaw *et al*., 2008; Llamas *et al*., 2012; Jenkins *et al*., 2013). The scarcity of raw material and the controversy arising from the use of soils for energy crops versus food crops are additional problems that must also be surmounted before this technology can be fully commercialized.

FT‐SPK is produced from pyrolysis of biomass to produce synthetic gas followed by Fischer‐Tropsch synthesis, hydroprocessing and separation (Hong *et al*., 2013; Kallio *et al*., 2014a,b). Following the classification devised by Wang and Tao (2016), this process is a GTJ fuel technology (Table 1). Any type of biomass can be used as feedstock (e.g., wastes, agricultural, and others); biomass is dried to reduce particle sizes and is converted to syngas. Syngas is then inputted into the Fisher‐Tropsch process, which comprises a series of catalytic steps to convert syngas into liquid hydrocarbons (Wang and Tao, 2016). The main advantages of this bio‐jet fuel are that it is free of sulfur and that it contains very few aromatics. FT‐SPK has also been approved by the ASTM and has been used for testing flights, however, the process is still at an early stage of technological development (Blakey *et al*., 2011).

Up until now, most of the bio‐jet fuels tested by commercial airlines or military flights have been produced by OTJ conversion. Most often, vegetable oils from feedstocks such as coconut, Jatropha and Camelina have been used, although waste cooking oil and algae have been also used (summarized by Wang and Tao, 2016). As mentioned, intensification of oily crop cultivation for bio‐fuel will compete with food‐crops, and is a currently a contentious subject. GTJ has not been extensively tested for commercial or military flights and, so far, only industrial waste gas and natural gas have been used as feedstock.

These pre‐commercial technologies are complex and require multiple‐steps—each additional step increases the production cost; thus, simplification of the process scheme will be beneficial for costs and will enable improved competitiveness with fossil fuels.

Alternatively, two additional technologies, alcohol‐to‐jet fuel (ATJ) and sugar‐to‐jet fuel (STJ) are under development (Table 1), although they are still a far away from market entry. ATJ has been mainly studied as a way to convert ethanol and butanol to jet fuels through alcohol dehydration, oligomerization and hydrogenation, while STJ involves the biological and catalytic conversion of sugars to hydrocarbons.

**Figure 1:**
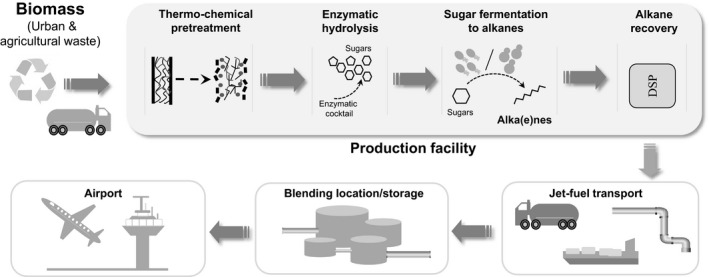
Schematic representation of the conversion of wastes (agricultural or from municipal solid wastes) to alkanes using 2G technologies and microorganisms. DPS, downstream processes.

The development of second generation (2G) bioethanol (production of bioethanol from lignocellulosic material) has paved the way for the industrial production of sugars from renewable sources such as agricultural or municipal wastes. Therefore, technologies for the conversion of sugars to hydrocarbons are a promising approach towards sustainable bio‐jet fuel production. This conversion can be done either by catalytically upgrading sugars (or its intermediates), or by biological fermentation (Wang and Tao, 2016; Fig. 1). The advantages are that fermentation of sugars to jet fuel does not require chemical catalysts, high energy or high temperature reactions, and the conversion can be carried out in a single fermentation tank, which reduces the number of production steps.

The remainder of this review will be centred on the STJ technology with a focus on the biological conversion of sugars to hydrocarbons. For the reasons listed above, this technology represents a highly promising option for the production of bio‐jet fuels. Achieving the synthesis of alkanes using microorganisms has challenged biotechnologists for years. Intensive research efforts are currently underway to identify microorganisms that are able to carry out these transformations, as well as to understand the complexity of related biochemical pathways, and to develop an economically and technologically feasible industrial process based on this conversion.

## Fermentation of sugars to alka(e)nes: state of the art

### Microbes that produce alkanes

**Figure 2:**
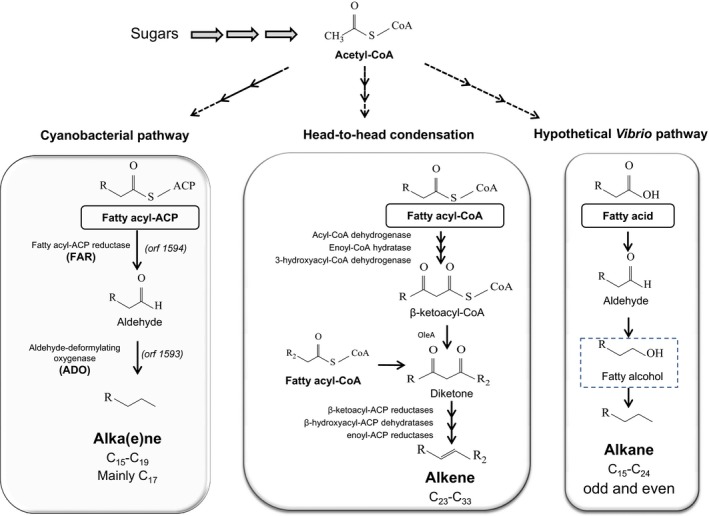
Schematic representation of the natural alkane biosynthesis pathways. Each pathway has different starting compound (in boxes). Enzymes of the cyanobacterial pathway (FAR and ADO) are depicted in bold as they have been used in different engineered bacteria. *orf1594* and o*rf1593* refers to the genes firstly identified in *Synechococcus elongatus* PCC7942 as involved in alkane biosynthesis. Production of alcohol in the hypothetical *Vibrio* pathway is depicted with a discontinuous line box as it is the main characteristic of this pathway.

The quest to find or construct a suitable microbial organism for the synthesis of alkanes from sugars intensified almost a decade ago, when the need for renewable biofuels was driven by increasing oil prices, the political instability associated with many producer countries, and when the reduction of GHG became a priority. However, there exist reports, from long before this interest flourished, demonstrating that cyanobacteria are able to synthesize alkenes and (linear and branched) alkanes. Studies made during 1960's and 1970's showed that cyanobacteria produced hydrocarbon in the suitable range for jet fuel (C_15_ to C_19_), with heptadecane being the predominant *n*‐alkane in most cyanobacteria species (Han *et al*., 1968, 1969; Gelpi *et al*., 1970; Blumer *et al*., 1971). The biological meaning of alkane production in cyanobacteria remains a mystery; deletion of genes do not have obvious growth‐related phenotypes (at least in the lab); and furthermore, 10% of the cyanobacterial strains that have been sequenced to date do not carry the required genes. It has been speculated that cyanobacteria produce these chemicals to influence membrane properties (i.e. fluidity) to adapt to certain (yet unknown) conditions (Klähn *et al*., 2014). Nevertheless, the cyanobacterial alkane biosynthesis pathway had been inferred: fatty‐acyl‐ACPs of even‐numbered carbons are converted into fatty aldehydes by the action of a fatty acyl‐ACP reductase (FAR) and these aldehydes are converted into an alkane or alkene with the concomitant loss of one carbon through of the action of an aldehyde decarbonylase (AD or ADC; latter named ADO) (Fig. 2). It was not until 2010 that the biochemical steps for alkane biosynthesis in cyanobacterias were demonstrated (Schirmer *et al*., 2010). Identification of the pathway was done through bioinformatic analysis, by assuming that alkane biosynthesis pathway was not present in the genomes of non‐producing cyanobacterial strains, and doing subtractive genome analysis with those that produced hydrocarbons. Seventeen genes were present in the producing strains that were not present in non‐producing strains. Of two with unknown function, one shared similarity with short‐chain dehydrogenases (e.g., *orf*1594 from *Synechococcus elongatus* PCC7942) and was identified as the FAR, while the second shared similarity with ferritin‐like or ribonucleotide reductase‐like proteins (e.g., *orf*1593 in *S. elongatus* PCC7942) and was identified as the AD (Fig. 2). Homologous genes were identified from several cyanobacteria, demonstrating that this pathway is present in most alkane‐producing cyanobacterial species (Schirmer *et al*., 2010). ADC had been previously studied in plants and algae, where the enzyme released CO from the fatty acid aldehyde (Dennis and Kolattukudy, 1992; Schneider‐Belhaddad and Kolattukudy, 2000). However, in cyanobacteria, formate is the co‐product of the reaction, and therefore the enzyme was re‐designated as an aldehyde‐deformylating oxygenase (ADO) (Li *et al*., 2012).

Some aerobic Gram‐positive bacteria, such as *Sarcina lutea* ATCC 533, synthesize long‐chain alkenes with at least one double bond and chain lengths between C_23_ to C_33_. The synthesis pathway of these alkenes is known as head‐to‐head condensation of fatty acids (Fig. 2) and consists of the formation of a carbon‐to‐carbon bond between the carboxyl carbon of one fatty acid and the α‐carbon of another fatty acid (Albro and Dittmer, 1969; Beller *et al*., 2010). This pathway begins with the formation of fatty acyl‐CoA (or ACP) that is converted to β‐ketoacyl‐CoA likely by the sequential action of an acyl‐CoA dehydrogenase, an enoyl‐CoA hydratase and a 3‐hydroxyacyl‐CoA dehydrogenase (from the early steps of the β‐oxidation pathway). Subsequently, the enzyme that mediates condensation, known as OleA, produces a diketone and finally, a series of reduction and dehydration reactions lead to alkene biosynthesis (Beller *et al*., 2010; Sukovich *et al*., 2010).

Although bacteria from the genus of *Vibrio*, were described as able to synthesize alkanes, being predominant heptadecane (Oró *et al*., 1967), these are controversial results. *Vibrio furnissii* M1, a halotolerant bacterium, was reported to synthesize alkanes between C_15_ and C_24_ (Park *et al*., 2001). This Eubacteria produces odd and even‐chain alkanes through a different process than cyanobacteria, which only produce even‐chain fatty acids. Park (2005) showed the production of *n*‐hexadecane when 1‐hexadecanol was fed to membrane preparations, proving that alkane can be derived from the corresponding alcohol. When 1‐[1‐^14^C]hexadecanoic acid was fed to the membrane preparation, the radioisotope label was identified in alcohol, aldehyde and alkane derivatives; this supported the hypothesis that the 1‐hexadecanoic acid is converted to 1‐hexadecanal and that this aldehyde can be transformed to pentadecane and carbon monoxide or to 1‐hexadecanol, which is further transformed to hexadecane (Park, 2005; Fig. 2). However, a genomic and biochemical study carried out with this strain report the lack of alkane‐producing phenotype (Wackett *et al*., 2007). This is in line with the fact that, after complete genome sequencing and annotation of a free‐living *Vibrio furnissii* sp. nov. strain (NCTC 11218), no obvious genes for the synthesis of alkanes were found (Lux *et al*., 2011).

Many other Eubacteria are able to synthesize alkanes, although most of them show the same limitations displayed by cyanobacteria: they only produce low amounts of alka(e)nes (in the order of 0.005–0.25% of dry biomass (Ladygina *et al*., 2006; Coates *et al*., 2014), and they produce them intracellularly and therefore are difficult to separate and purify from the cells (Jansson, 2012; Fu *et al*., 2015). In cyanobacteria alka(e)nes are usually found in lipid droplets (LD), packed with several hydrophobic energy‐dense compounds surrounded by a lipid mono‐layer (Peramuna and Summers, 2014). Only few prokaryotes can produce intracellular and extracellular hydrocarbons; among these, anaerobic bacteria from the genus *Clostridium* and *Desulfovibrio* have shown the most promising results. These bacteria produce intracellular and extracellular hydrocarbons between C_11_ and C_35_ and predominantly long‐chain *n*‐alkanes from C_25_ to C_35_ (Han and Calvin, 1969; Bagaeva and Zinurova, 2004). It has been hypothesized that in the sulfate‐reducing bacteria *Desulfovibrio desulfuricans*, acetate and formate generated from CO_2_ are reduced to aldehydes, which, in turn, undergo aldol condensation that elongate the carbon chain to produce hydrocarbons.

The great advantage of using cyanobacteria in industry is that this is a photosynthetic microorganism, so they can synthesize alkanes from sunlight and CO_2_ (Lau *et al*., 2015). However, despite the interest in all the natural alkane producing microorganisms, it is clear that production levels are not industrially relevant. Therefore, different methods and genetic modifications to improve production have been investigated (Table 2). Some studies have focused on improving alkane production by modification of growth and environmental conditions. The nitrogen‐fixing cyanobacterium *Anabaena* sp. PCC7120 is able to double its intracellular alkane production when it was exposed to nitrogen deficiency or salt stress (Kageyama *et al*., 2015). Peramuna *et al*. (2015) combined genetic modifications and cultivation conditions to demonstrate that cultivation of *Nostoc punctiforme* cells that overexpressed the genes that encode the native FAR, ADO and a putative lipase (Npun_F1710/11 and Npun_F5141) under high light conditions increased alka(e)ne accumulation in LD by more than 11.5 fold (from 1.12% to 12.9% of CDW).

**Table 2:** Main properties of the alkane‐producing strains

Strain	Modifications	Amount of produced alka(e)nes	Alka(e)nes chain	Reference
Cyanobacterias				
*Anabaena* sp. PCC7120	Exposition to nitrogen deficiency or salt stress	1200 μg g^−1^ CDW	C_17_	Kageyama *et al*. (2015)
*Nostoc punctiforme* PCC 73102	Overexpression of several copies of FAR and ADO (Npun_F1710/11) and a lipase (Npun_F5141) under high light conditions	12.9% of CDW	C_17_	Peramuna *et al*. (2015)
*Synechocystis* sp. PCC6803	Overexpression of the native acetyl‐CoA carboxylase (*accABCD*), the lipase *lipA*, the acyl‐ACP synthetase *slr1609*, the native FAR and ADO and deletion of the β‐ketolyase *phaA*	6.5 mg l^−1^ (1.3% CDW)	C_17_	Wang *et al*. (2013)
*Escherichia coli*				
*E. coli* MG1655	Expression of FAR from *Synechococcus elongatus* PCC7942 and ADO from *N. punctiforme* PCC73102 and use a modified medium	300 mg l^−1^	C_13_, C_15_ and C_17_	Schirmer *et al*. (2010)
*E. coli* BL21(DE3)	Expression of FAR and ADO from *S. elongatus* PCC7942 and overexpression of the transcription factor *fadR* and aldehyde reductase, *yqhD*	255.6 mg l^−1^	C_15_ and C_17_	Song *et al*. (2016)
*E. coli* BL21(DE3)	Expression of FAR and ADO from *S. elongatus* PCC7942 and *fabH2* from *Bacillus subtilis*	98.3 mg l^−1^	C_13_, C_14_, C_15_, C_16_ and C_17_	Harger *et al*. (2013)
*E. coli* BL21(DE3)	Expression of reductase complex from *Photorhabdus luminiscens* (*luxcED*) and ADO from *N. Punctiforme*	~8 mg l^−1^	C_13_, C_15_, C_16_ and C_17_	Howard *et al*. (2013a,b)
*E. coli* W3110	Expression of an acyl‐CoA reductase from *Clostridium acetobutylicum* (*acr*) a fatty aldehyde decarbonylase from *Arabidopsis thaliana* (CER1), deletions in *fadE* and *fadR*, overexpression *fadD* and a modified version of a thioesterase ‘TesA[L109P]	580.8 mg l^−1^ (in 2.1 l fed‐batch fermentation)	C_9_, C_12_, C_13_, and C_14_	Choi and Lee (2013)
*E. coli* BL21(DE3)	Overexpression an acyl‐ACP thiosterase from *Umbellularia californica* (UcFatB), the native fatty acyl‐CoA synthetase (FadD), a fatty acyl‐CoA reductase from *Acinetobacter* sp. M‐1 (AcrM), and an aldehyde decarboxylase from *N. punctiforme*	4.04 mg g^−1^ CDW	C_11_ and C_13_	Yan *et al*. (2016)
*E. coli* BL21(DE3)	Co‐expression of the thiosterase TesA, a carboxylic acid reductase (CAR) from *Mycobacterium marinum*, a phosphopantetheinyl transferase (Sfp) from *B. subtilis* and an aldehyde decarbonylase (ADC) from *Prochlorococcus marinus*	~2 mg l^−1^	C_11_, C_13_, C_15_ and C_17_	Akhtar *et al*. (2013)
*E. coli* MG1655	Reverse‐β‐oxidation pathway and overexpression of a CAR from *Nocardia iowensis* and an aldehyde reductase (AD) from *Prochlorococcus marinus*	~1.4 mg l^−1^	C_4_ and C_5_	Sheppard *et al*. (2016)
*E. coli* BL21(DE3)	Expression of AAR and ADO from *S. elongatus* PCC7942. Overexpression of the electron transfer system Fd/FNR from *S. elongatus* PCC7942	1.31 g l^−1^ (0.01 g g^−1^ CDW) in 2.5 l fed‐batch fermentation (101.7 mg l^−1^ in glass tubes)	C_15_ and C_17_	Cao *et al*. (2016)
Yeasts				
*Saccharomyces cerevisiae INVSc1*	Expression of aldehyde decarbonilase (CER1) and wax‐associated protein (CER3) from *Arabidopsis thaliana*; mutation in the yeast elongase component SUR4	19 μg g^−1^ CDW	Mainly C_29_	Bernard *et al*. (2012)
*S. cerevisiae* CEN.PK 113–11C	Expression of FAR and ADO from *S. elongatus* PCC7942, overexpression of the *E. coli* ferredoxin and the ferredoxin NADP^+^ reductase (*Fdx* and FNR) and deletion of *hfd1*	22 μg g^−1^ CDW	C_13_, C_15_ and C_17_	Buijs *et al*. (2015)
*S. cerevisiae* BY4741	Overexpression of a fatty acid alpha‐dioxygenase (alphaDOX) from *Oryza sativa*; mutations in the *faa1* and *faa4* genes	~70 μg l^−1^	C_14_ and C_16_	Foo *et al*. (2016)
*S. cerevisiae* CEN.PK 113–11C	Construction a chimeric citrate lyase; overexpression of exogenous FFA synthases and endogenous acetyl‐CoA carboxylase; mutations in the alcohol dehydrogenase (Adh5p) and in several genes which can consume FFA (*faa1∆*,* faa4∆*,* pox1∆* and *hfd1∆*); expression of CAR from *Mycobacterium marinum* and an ADO from *N. punctiforme*	0.82 mg l^−1^	C_13_, C_15_ and C_17_	Zhou *et al*. (2016)
*Yarrowia lipolytica* PO1F	Overexpression of a lipoxygenase from soybean (Gmlox1); mutation of *mfeI* to block the β‐oxidation pathway	4.98 mg l^−1^	C_5_	Blazeck *et al*. (2013)
*Aerobasidium pullulans* var *melanogenunm*	Wild‐type	21.5 g l^−1^	C_14_, C_26_, C_27_ and C_28_	Manitchotpisit *et al*. (2011)
*Cupriavidus necator* H16	Deletion of the *phaCAB* operon and overexpression of the FAR and ADO from *S. elongatus* PCC7942	435 g l^−1^	C_15_ and C_17_	Crépin *et al*. (2016)

Since the pool of precursors needed to synthesize alka(e)nes are fatty acyl‐ACPs, the strain *Synechocystis* sp. PCC6803 was genetically modified to redirect the carbon flux towards them. In order to achieve this increased pool, the authors overexpressed the native acetyl‐CoA carboxylase (*accBCDA*), which catalyses the initial step in the fatty acid biosynthesis pathway, the *lipA* gene which releases FFAs from membrane lipids and the acyl‐ACP synthetase (*slr1609*), which transforms FFAs into acyl‐ACP. In addition, the deletion of the β‐ketolyase *phaA* (*slr1993*) prevented the formation of poly‐β‐hydroxybutyrate (PHB), a reservoir compound that competes with alkanes for available carbon. These modifications, together with the overexpression of the native FAR and ADO enzymes enabled alka(e)ne production at rates that were 8.3 times higher than in the wild‐type (Wang *et al*., 2013). Interestingly, this increase was observed when the genes that encode FAR and ADO were introduced into two different loci (*slr0168* and *slr1556*), illustrating the importance of the level of expression of the reductase and decarbonylase genes in alka(e)ne production (Wang *et al*., 2013).

Regardless all the improvements to date, the final amount of alka(e)nes produced by cyanobacteria is limited to few milligrams per 100 g of CDW and therefore production improvements or the identification of new host organisms are required. Industry has displayed clear preferences for certain microorganisms as production platforms (e.g., *Escherichia coli*,* Bacillus subtilis*,* Aspergillus nidulans* or the yeast, *Saccharomyces cerevisae*) and several of these microorganisms have been tested for alka(e)nes production (Table 2). Despite this preference, new platform candidates are currently under examination due to potential production capabilities or improved suitability for industrial alkanes processes.

### Engineering industrial bacterial platforms for the production of alkanes

One of the first approaches towards developing high volume synthesis of alkanes was to express the cyanobacteria pathway in *E. coli*. Expression of FAR and ADO from *S. elongatus* PCC7942 gave production of around 25 mg l^−1^ of pentadecane and heptadecene among other alcohols and aldehydes. Replacing the *S. elongatus* ADO with that of *N. punctiforme* PCC73102, increased the yield up to 80 mg l^−1^ of tridecane, pentadecene, pentadecane and heptadecene. Alkane titres were improved up to 300 mg l^−1^ when a modified medium was used (Schirmer *et al*., 2010). This method to produce alkanes and alkenes is patented by the company LS9 (Schirmer *et al*., 2014).

**Figure 3:**
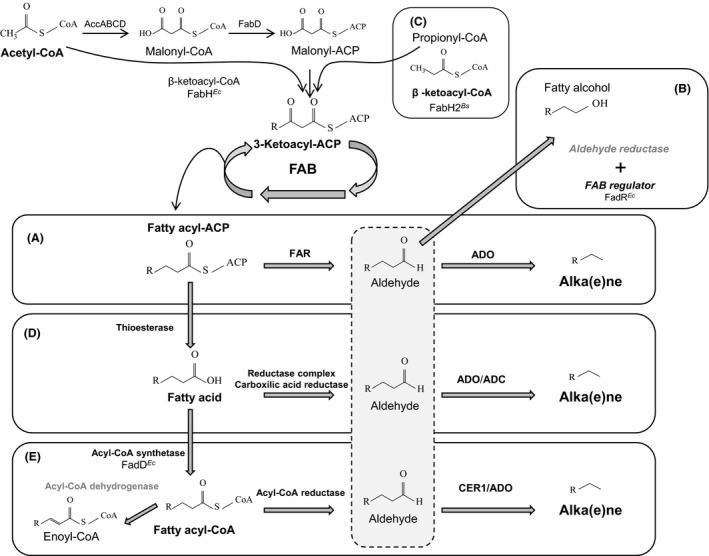
Schematic representation of different recombinant pathways incorporated in microorganisms that led to alkane biosynthesis. (A) The *S. elongatus* pathway to synthetize alkanes from acyl‐CoA, as in Schirmer *et al*., 2010; (B) Fatty alcohols are concomitantly produced from aldehydes, a sidetrack that could be partially blocked mutating aldehyde reductases and overexpressing of FadR, as in Song *et al*., 2016; (C) Overexpression of FabH2 from *B. subtilis* allows wider range of alkanes when cultivated in propanoate (Harger *et al*., 2013); (D) Alkanes are also produced directly from fatty acids (Howard *et al*., 2013a); (E) but also from fatty acyl‐CoA, which requires several genetic modifications to improve fatty acyl‐CoA accumulation (Choi and Lee, 2013; Steen *et al*., 2010 and Yan *et al*., 2016); Bold letters indicate gene overexpression; grey letters indicate knocked‐out mutants; FAB, Fatty acid biosynthesis; Ec, *E. coli*; Bs, *B. subtilis*; ADO, Aldehyde deformylating oxigenase; ADC, Aldehyde decarboxylase.

With the aim of increasing the amount of precursors available for alkane biosynthesis, efforts have been mostly directed towards the improvement of expression of the FAR and ADO genes and towards the re‐direction of fatty acid metabolism carbon flux to fatty acyl‐ACP (Fig. 3A). Expression of different cyanobacteria FAR and ADO genes, utilization of different expression vectors, induction conditions and culture media have been tested in numerous laboratories (Schirmer *et al*., 2010; Choi and Lee, 2013; Cao *et al*., 2014; Song *et al*., 2016). Depending on the *E. coli* strain and plasmids used, the results were variable and it is difficult to reach a consensus about how to improve yield on the basis on this diverse set of experiments. However, it is clear that increasing carbon flux towards fatty acyl‐ACP synthesis is a good approach to improve alka(e)ne biosynthesis. Song *et al*. (2016) demonstrated that overexpression of *fadR*, a transcription factor that controls the expression of several genes involved in fatty acid biosynthesis, degradation and transport through the membrane (Zhang *et al*., 2012), increased alkane production from 24 to 53.4 mg l^−1^ in the parental strain. However, overexpression of other genes involved in fatty acid biosynthesis such as *fabH* or *fabB* did not improved alka(e)ne biosynthesis. The combination of *fadR* overexpression and the mutation of a gene encoding an endogenous aldehyde reductase, *yqhD*, improved production up to 255.6 mg l^−1^. YqhD is the enzyme responsible for the conversion of fatty aldehyde into fatty alcohols and as such is an enzyme that competes for the same substrate with ADO (Song *et al*., 2016; Fig. 3B).

There are other recombinant *E. coli* cells capable of producing a different range of alkanes (tridecane, tetradecane, pentadecane, hexadecane, heptadecene and heptadecane) (Harger *et al*., 2013). In order to modify the product profile of the cyanobacteria alkane biosynthetic pathway, *E. coli* required the expression of *fabH2* from *B. subtilis* in addition to the expression of the FAR and ADO enzymes from *S. elongatus*. *E. coli* FabH catalyses the condensation between an acyl‐CoA (usually acetyl‐CoA) and malonyl‐CoA in the initiation step of fatty acid biosynthesis, producing even‐chain length fatty acid (Fig. 3). However, the *B. subtilis* variant, FabH2, presents higher levels of activity on acyl‐CoAs that have chain lengths longer than two carbons, this resulted in a wider profile of alkane production when the cells are cultivated with propanoate. The final strain with FAR, ADO and FabH2 produces 98.3 mg l^−1^ of alkanes and alkenes (Harger *et al*., 2013; Fig. 3C).

There are alternative synthetic pathways for production of alka(e)nes that use fatty acyl‐CoA or FFAs as starting substrates, which are later converted to fatty aldehydes (Steen *et al*., 2010). Production of alka(e)nes directly from FFA was achieved by expressing the reductase complex from *Photorhabdus luminiscens* (composed of *luxC*, a fatty acyl reductase, *luxE*, a fatty acyl synthetase, *luxD*, a fatty acyl transferase), and an ADC from *N. punctiforme* (*Np*AD) (Fig. 3D). The engineered cells can produce tridecane, pentadecene, pentadecane, hexadecene, heptadecene and heptadecene in a total yield of ~8 mg l^−1^ (Howard *et al*., 2013a). In this case production of fatty alcohols and traces of fatty aldehydes was also observed. By increasing the FFA pool (modifying the native pathways or adding exogenous fatty acids) it is possible to change the composition of the synthesized alkanes (Howard *et al*., 2013a). The Shell Oil Company has patented this last pathway (Howard *et al*., 2013b).

By generating an *E. coli* strain with deletions in *fadE* and *fadR* that also overexpressed *fadD* and a modified version of a thioesterase gene, Choi and Lee (2013) reported production levels of short‐chain alkanes (nonane, dodecane, tridecane, 2‐methyl‐dodecane and tetradecane) of 580.8 mg l^−1^ from acyl‐CoAs (Fig. 3E). *fadE* encodes an acyl‐CoA dehydrogenase that catalyses the first step of the β‐oxidation pathway and therefore the deletion mutant cannot degrade fatty acids, while deletion of the regulatory gene, *fadR*, increased the production of short‐chain FFA. Thioesterases are enzymes that catalyse the transformation of fatty acyl‐ACP to FFAs and the modified version (‘TesA[L109P]) used in this strain lacks the signal peptide for secretion making it more active than native TesA. Overexpression of the *fadD* gene improves the conversion of FFAs into fatty acyl‐CoA. In this case, the pathway used for alka(e)ne production was a combination of the fatty acyl‐CoA reductase from *Clostridium acetobutylicum* (*acr*) and a fatty ADC from *Arabidopsis thaliana* (CER1) which produces short‐chain alkanes. Despite the relatively good production levels (580 mg l^−1^), short‐chain fatty alcohols as well as trace amounts of fatty aldehydes were also detected (Choi and Lee, 2013). A high level production of medium‐chain alkanes has been achieved in *E. coli* (2.21 mg g^−1^ CDW of undecane and 1.83 mg g^−1^ CDW of tridecane) through the overexpression of (i) an acyl‐ACP thiosterase from *Umbellularia californica* (UcFatB), which preferentially acts on lauroyl‐ACP, (ii) the native fatty acyl‐CoA synthetase (FadD); (iii) a fatty acyl‐CoA reductase from *Acinetobacter* sp. M‐1 (AcrM) that prefers lauroyl‐CoA and myristoyl‐CoA as substrates, and (iv) an aldehyde decarboxylase from *N. punctiforme* (Yan *et al*., 2016; Fig. 3E).

Many other enzymes have been tested for alkane production in *E. coli*, although they tend to yield lower amounts of product. For example, co‐expressing the *E. coli* thiosterase TesA, a carboxylic acid reductase (CAR) from *Mycobacterium marinum* (which activates and reduces the FFA to aldehyde), a phosphopantetheinyl transferase (Sfp) from *B. subtilis* (for CAR enzyme activation) and an ADC from *Prochlorococcus marinus*, allows the recombinant cells to produce ~2 mg l^−1^ of undecane, tridecane, pentadecene and heptadecene (Akhtar *et al*., 2013).

**Figure 4:**
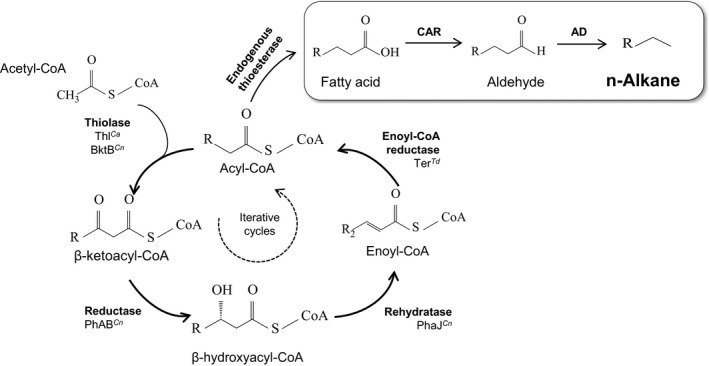
Schematic representation of the production of alkanes via reversed β‐oxidation used by Sheppard *et al*. (2016). Cu, *Cupriavidus necator*;* Ca*,* Clostridium acetobutylicum*;* Td*,* Treponema denticola*.

*Escherichia coli* recombinant strains have also been used to test pathways to produce short‐length alkanes, such as propane (Kallio *et al*., 2014b), butane, pentane, heptane or nonane (Sheppard *et al*., 2016). C_4_ to C_9_ alkanes are major components of gasoline, and Sheppard *et al*. explored different alternatives to generate these short‐length alkanes. They demonstrated the advantages of the reverse‐β‐oxidation (RBO) pathway for the synthesis of short‐chain alkanes (Fig. 4). The RBO, proposed previously for the synthesis of long‐chain alcohols and carboxylic acids (Dellomonaco *et al*., 2011), presents some advantages compared with the fatty acid synthesis pathway (FAS) when it comes to supplying the necessary acyl substrates. On the one hand, it is more balanced in terms of ATP consumption and cofactor utilization, since it only requires NADH for chain elongation, and not NADPH which is required by the FAS pathway. In addition, the primary substrate for the RBO pathway is acetyl‐CoA directly, whereas FAS requires the synthesis of malonyl‐ACP, which also consumes ATP and ACP. However, the utilization of this pathway to generate the hydrocarbons required for bio‐jet fuel (C_9_ to C_16_) would require an increasing number of RBO cycles in order to obtain the longer chains; as such, to the best of our knowledge, the production of fatty acids or alkanes with chains longer than C_10_ has not yet been achieved.

It is worth noting that in most of the studies described above alcohols are a concomitant product of alkane synthesis, a byproduct which decreases the potential production yield. Although alcohols are produced due to endogenous *E. coli* aldehyde reductases converting fatty aldehydes in fatty alcohols (Zheng *et al*., 2012), an additional route occurs via the enzyme ADO, which besides catalysing the alkane synthesis through the release of formate, is also able to catalyse the incorporation of an oxygen atom from O_2_ into the alkane product to generate alcohol and aldehyde products. This reaction, which has been demonstrated in vitro (Aukema *et al*., 2013), does not rule out the participation of existing *E. coli* aldehyde reductases in the formation of alcohol, as has been demonstrated in several papers (Kallio *et al*., 2014b; Rodriguez and Atsumi, 2014; Kunjapur and Prather, 2015). It may be possible that the enzyme ADO favours one product over another in vivo, or even that these preferences are affected depending on the surrounding environment, such as the strain background used to overexpress it. Deleting the putative aldehyde reductases works to reduce alcohol production, however, it would also be desirable to find ADO from different organisms, or develop modified, fine‐tuned versions of the known ADO enzymes with enhanced ADC activity that direct the substrate towards alkanes instead of alcohols.

Nevertheless, the negative role of alcohol production during alkane biosynthesis has been questioned very recently (Cao *et al*., 2016). In fact, due to the low activity demonstrated by ADO and the negative impact of aldehyde accumulation for the cell, a certain level of alcohol production helps ADO to balance the equilibrium between aldehyde formation and conversion. By testing multiple levels of expression of exogenous FAR, ADO and the aldehyde reductase (*adhP*) the authors concluded that alkanes absolute production is enhanced by reaching an optimized ration between alkanes and alcohols, instead of eliminating alcohols completely. In addition, in order to increase the amount of precursors, the authors affected the metabolic flows in several ways. Importantly, driving carbon towards acetate did not have any effect, but it positively did to optimize the electron transfer system through the expression of a reducing system, formed by 2Fe–2S ferredoxin (Fd) and its counterpart ferredoxin‐NADP^+^ reductase (FNR), from *S. elongatus* PCC7942. This supply is necessary for maximizing cyanobacterial ADO activity, and the reason behind this strategy is because a peroxo‐intermediate (H_2_O_2_) is formed when the aldehydes are transformed to alkanes (Pandelia *et al*., 2013; Paul *et al*., 2013) which can inhibit ADO activity (Andre *et al*., 2013). With these modifications, the produced alkanes increased from 58.8 to 101.7 mg l^−1^, and what is more, they achieved 1.31 g l^−1^ alkanes (pentadecane, pentadecene and heptadecene) in 2.5 fed‐batch fermentation, the highest reported titre of alkanes in any recombinant organism until now.

### Engineering yeast for the production of alkanes

The yeast *Saccharomyces cerevisiae* has a predominant role in the biotechnological industry. The food industry has developed fermentation technologies using yeast for hundreds of years, and this has contributed to the selection of an extremely robust, adaptable and tolerant organism that is useful for industrial processes. Beyond any doubt, these advantages, together with the powerful genetic tools developed for its manipulation have made *S. cerevisiae* a very attractive organism when it comes to designing new biotechnological industrial processes. This is particularly true for companies interested in biofuel production as they have previously developed facilities to produce bioethanol and they possess the know‐how for manipulation of yeast. As summarized in Hong and Nielsen (2012), there are a multitude of examples that demonstrate the use of yeast for the synthesis of a broad spectrum of chemicals of interest. Nevertheless, the examples using yeast to produce alkanes are quite limited, and one thing they have in common is that the amounts produced by *S. cerevisiae* are clearly insufficient (Table 2).

Bernard *et al*. (2012) described the first recombinant *S. cerevisiae* that produced alkanes; although this work focused primarily on the functional and biochemical characterization of the CER1 protein from *Arabidopsis thaliana* and not on the potential use of the yeast as an alkane production tool. CER1, which was previously implicated in alkane synthesis in plants (Aarts *et al*., 1995), acts specifically as an ADC when it is expressed in yeast, in partnership with a second protein, CER3. Both peptides work together to produce long chain alkanes, mainly nonacosane (C_29_), which requires very long fatty acids as a substrate. Thus, alkanes are only accumulated in a *sur4* F262K/K266L yeast mutant background (a mutation in an elongase that allows the synthesis of C_28_ and C_30_ fatty acids), although the productivity was around 19 μg g^−1^ of CDW. The caveat of using these very long alkanes for biofuels is their high melting point and low solubility; their function in the plant is to produce protective waxes. Nevertheless, this work did not study alkanes from a biotechnological perspective, and the potential use of this pathway in yeast has not been extensively explored. In contrast, the cyanobacterial pathway used previously in *E. coli* (Schirmer *et al*., 2010) has also been tested in *S. cerevisiae* (Buijs *et al*., 2015). The acyl‐ACP reductase and the aldehyde deformylating enzymes *Se*FAR and *Se*ADO were overproduced in yeast, and because the endogenous proteins necessary to supply reducing power to the ACP reductase are enclosed in the mitochondria, the *E. coli* ferredoxin and the ferredoxin NADP^+^ reductase (*Fdx* and FNR) were additionally expressed. Once again, the authors found serious restrictions in the production of alkanes which required modifications in the yeast background. They found that Hfd1p, a yeast aldehyde dehydrogenase, is able to oxidize the aldehyde intermediates to fatty acids. As such, knocking down *hfd1* in combination with overexpression of the alkane pathway, allowed the production of alkanes (22 μg g^−1^ CDW, mainly C_16_) but also alcohols (0.52 mg g^−1^ CDW, mainly C_15_). The fact that alcohols are produced preferentially suggests not only that the deformylating enzyme which transforms aldehydes to alkanes is a major bottleneck of the process, but also that, as in the case of bacteria, yeasts possess alternative enzymes that rapidly transform aldehydes into alcohols. These findings cannot, however, rule out the hypothesis of an alternative aldehyde dehydrogenases, other than *hfd1*, that might also affect alkane productivity.

An alternative tactic to improve the synthesis of alkanes in *S. cerevisiae* has combined several approaches (Foo *et al*., 2016). Firstly, the authors proposed FFA instead of glucose as a substrate to synthesize alkanes, since glucose requires the formation of fatty acyl‐CoA to enter into the pathway and this is a strongly regulated process. And secondly, they overexpressed a fatty acid alpha‐dioxygenase (alphaDOX) from *Oryza sativa* (rice) to produce aldehydes from fatty acids, which uses dioxygen as a cofactor and does not compete for the cellular reducing power of NADPH. This enzyme oxidizes fatty acids to form 2‐hydroperoxides that spontaneously form the C_n‐1_ aldehyde. The idea behind this proposal was to boost the accumulation of aldehydes, which seems to be a limiting step. For this, the yeast also required mutations in the *faa1* and *faa4* genes that would recycle the FFA to fatty acyl‐CoA. In addition, in order to enhance yeast access to the FFA the pH is a critical limiting step that demands adjustment. The authors observed production of alkanes (~70 μg l^−1^) and alcohols (~1500 μg l^−1^), demonstrating again the importance of the existing strong aldehyde reductases in yeast. Technologically speaking, the major issue of this proposed method would be to provide FFA to the yeast, which would increase costs to an industrial design compared with the direct use of sugars. Thus, a similar strategy, using FFA accumulation to produce alkanes, has been recently advanced, but this time providing glucose (Zhou *et al*., 2016) and modifying the yeast background profusely in order to accumulate FFA and produce either alkanes or fatty alcohols from them. In order to achieve accumulations of 10.4 g l^−1^ of FFA, which according to the authors is the highest FFA titre ever reported in *S. cerevisiae*, the authors tackled several pathways intensively. First, they constructed a chimerical citrate lyase pathway to synthesize more acetyl‐CoA by overexpressing foreign and modified versions of host genes, including a mitochondrial citrate transporter to export it to the cytoplasm. Second, they overexpressed exogenous FFA synthases with a higher activity profile than the native one, in addition to the endogenous acetyl‐CoA carboxylase to produce more malonyl‐CoA. And third they blocked sidetrack flows where the FFA could be consumed with several mutations (*faa1∆*,* faa4∆*,* pox1∆* and *hfd1∆*). Then, alkanes were produced by incorporating a CAR from *Mycobacterium marinum* and an ADO from *N. punctiforme* (which works better than ADO from *S. elongatus*). Even with a mutation in the alcohol dehydrogenase (Adh5p) which produces an excess of sided‐product fatty alcohols, the final outcome were 0.82 mg l^−1^ of alkane and 5 mg l^−1^ of alcohols. Whilst these could the highest alkane yields reported in *S. cerevisiae*, they are nowhere near sufficient to be industrially viable. Despite the magnitude of the modifications carried out in this strain, the authors surprisingly, did not consider how much of the glucose is driven directly towards ethanol through fermentation and how much FFA synthesis could be improved by short‐circuiting the major highway of carbon flux in yeast. Indeed, regardless of the FFA production levels reached, the authors concluded that this is not more than 10% of the theoretical yield and despite the efforts to improve the background strain, the lack of a competent producer strain is likely due to the bottleneck caused by the inefficient enzymes in charge of alkane synthesis.

**Figure 5:**
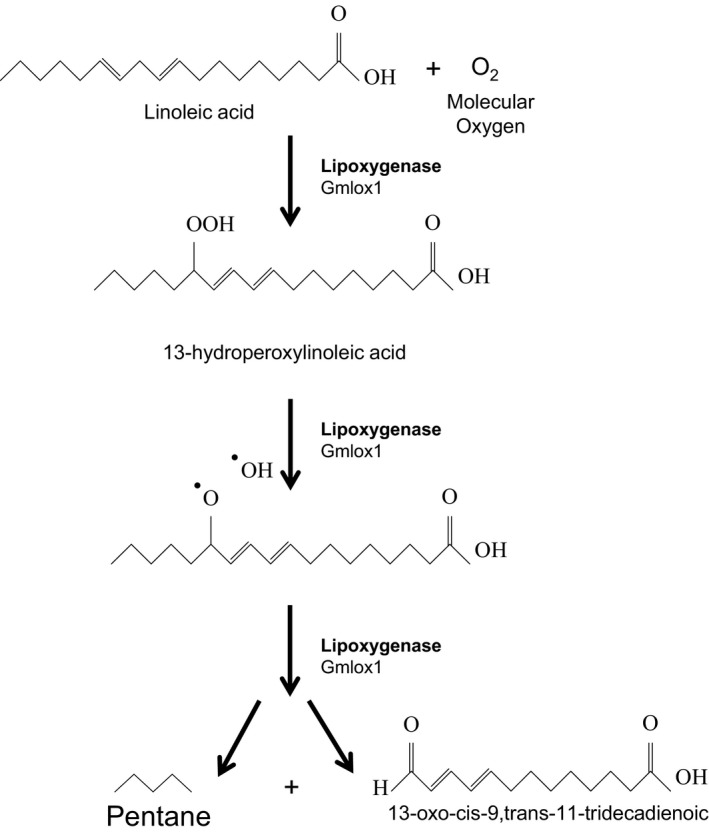
Schematic representation of the production of short chain alkanes in *Y. lypolytica* (modified from Blazeck *et al*., 2013).

Beyond the attempts to use *S. cerevisiae* to produce alkanes and other biofuels, alternative yeast model organisms have attracted the interest of the biotech community. Since the major prime material for alkane synthesis are fatty acids, a yeast that can produce large amounts of lipids is quite desirable, and this is the case for the oleaginous yeast *Yarrowia lipolytica*. This microorganism is able of accumulating 36% of CDW as lipids, and in acknowledgment of its potential as an industrial fuel producer (Beopoulos *et al*., 2009), a broad amount of research has focused on modifying *Y. lipolytica* to increase its lipid production (Beopoulos *et al*., 2011; Sabirova *et al*., 2011; Wang *et al*., 2011; Beopoulos *et al*., 2012; Rutter *et al*., 2015). In fact, a recent study succeeded in constructing a *Y. lipolytica* yeast through evolutionary engineering that accumulates a total of 39.1 g l^−1^ of lipid material (which is 76% of the theoretical maximum production and 87% of CDW as lipid content) (Liu *et al*., 2015). Despite this success in producing *Y. lipolytica* strains with high lipid content, the major drawback of this organism is its ability to grow with n‐alkanes as a carbon source. Its genome encodes 12 *alk* genes involved in the terminal mono‐oxygenation of n‐alkanes, each of them with different specificity according to alkane length (Iwama *et al*., 2016). Using this strain to synthesize alkanes would require further investigation into their alkane degradation profile and a focus on which of these *alk* genes must be removed to block the degradation pathway. That aside, Blazeck and colleagues managed to construct a strain able to synthesize the very short chain alkane pentane, which is also a component of jet fuels and gasoline (Blazeck *et al*., 2013; Fig. 5). These short chain alkanes are produced through a lipoxygenase instead of reduction and decarbonylation of fatty acids. Specifically, a lipoxygenase from soybean (Gmlox1) was overexpressed in *Y. lipolytica*, transforming linoleic acid into pentane and tridecadieonoic acid. However, despite optimization of the media for lipid synthesis and blocking the β‐oxidation pathway with a *∆mfeI* mutation to avoid consumption of linoleic acid, the pentane accumulation did not reach concentrations beyond 4.98 mg l^−1^. This suggests that regardless the enormous expectations for *Y. lipolytica*, much optimization will be required before it will be suitable to produce alkanes at an industrial scale.

*Aerobasidium pullulans* var *melanogenunm*, a yeast‐like fungus also known as black yeast due to their melanin production, can also accumulate important amounts of heavy oils (Manitchotpisit *et al*., 2011). A good example of this is the strain P10, which can accumulate up to 66.3% of CDW as lipids, the lipids mainly consist of palmitic (26.7%), oleic (44.5%) and linoleic acids (21%) (Wang *et al*., 2014). Importantly, Liu *et al*. (2014b) have reported a different strain of *A. pullulans* var *melanogenum* isolated from mangroves that is able to secrete up to 32.5 g l^−1^ of its heavy oils to the media, and most notably, 66.15% of the heavy oils were n‐alkanes, mainly tetradecane, hexacosane, heptacosane, octacosane. The authors propose a ‘head‐to‐head’ condensation mechanism behind the synthesis of these large amounts of alkanes, but there has been no biochemical characterization of the process; obviously due to its unique alkane production capacity, it would be of enormous interest to learn about the enzymatic players involved in this organism. The interest for industry also comes from the fact that the alkanes are completely secreted from the cell, which makes it much easier to extract from the production media. There appears to be promising industrial possibilities *for A. pullulans* regarding the production of biofuels. The strains described above are recent wild isolates, and as such, more focused research is necessary to find the most suitable fermentation conditions, and to learn if genetic alterations and strain improvements could enhance productivity.

Two recent reviews have focused on confronting the major existing limitations in using *S. cerevisiae* for the synthesis of alkanes among other bio‐fuels, underscoring the importance that this microorganism has as biotechnological workhorse (Lian and Zhao, 2015a; Sheng and Feng, 2015). A common message of both reviews is that the fatty acid metabolism, in general, and more specifically the synthesis of acetyl‐CoA is strictly regulated and not produced in sufficient amounts in yeast. This comes as no surprise considering that *S. cerevisiae* has been evolutionarily selected for its robust and efficient alcoholic fermentation capabilities. Thus, fermentation is a highly favoured process, which derives acetaldehyde produced from pyruvate into ethanol, and not into acetate and acetyl‐CoA, a reaction that is also heavily regulated in yeast (Lian and Zhao, 2015a). Funnelling the carbon stream towards acetyl‐CoA synthesis instead of ethanol has been strategically tackled as a way to increase FAS. Together with the overexpression of different heterologous genes that facilitate the synthesis of acetyl‐CoA, several studies have attempted to reduce the activity of the seven ADH enzymes, although the results vary according to which and how many of them are mutated, and which yeast strain used (Lian and Zhao, 2015b).

A different issue to consider is the limitation and imbalance of cofactors such as NADPH, which is mainly synthesized in the mitochondria and peroxisome, while the FAS occurs in the cytosol (Sheng and Feng, 2015). Hence, compartmentalization between different organelles and the cytosol must also be addressed as an important issue to solve. For example, a noteworthy solution that has successfully improved a biosynthesis process was implemented in the case of isobutanol production, by translocating the ketoisovalerate pathway (partly in charge of isobutanol formation) out of the mitochondria (Boles *et al*., 2009; Brat *et al*., 2012). This study proves that compartmentalization can be tackled to improve yield. The role of alkane tolerance in yeast has also been addressed, since no major production levels could be expected in an organism lacking sufficient resistance. C_9_ to C_11_ alkanes are highly toxic for *S. cerevisiae*. Transcriptome analysis revealed efflux pumps as a necessary mechanism for tolerance (Ling *et al*., 2013), and indeed, point mutations in transcriptional factors Pdr1p and Pdr3p that up‐regulate efflux pumps enhance yeast tolerance to C_10_ and C_11_ alkanes (Ling *et al*., 2015). In addition, acyl‐ACP reductases used to produce alkanes in bacterial recombinant systems have a preference for acyl‐ACP, which in yeast are mainly sequestered by the FAS, a large multi complex polypeptide protein system (FAS type I), instead of the bacterial FAS type II, mono‐functional proteins with lesser amount of constrained substrates (Lian and Zhao, 2015a).

### Production of alkane intermediates or of other hydrocarbons of interest

Isoprenoids (also named terpenoids or prenol lipids) are compounds derived from five‐carbon isoprene units, and consist of two or more hydrocarbon units arranged in different patterns; because they vary in the number of units and unit arrangements, they constitute a wide family of compounds. These compounds play multiple physiological roles in plants, animals and microbes and have numerous biotechnological applications (Boronat and Rodríguez‐Concepción, 2014; Islam *et al*., 2015). For example, squalene, an intermediate in cholesterol synthesis (Do *et al*., 2009), carotenoids which are responsible for red and yellow pigments in plants and microbes (Rivera Vélez, 2016), phytol the building block of chlorophyll (Mach, 2015), or the polyisoprenoid rubber, are examples of well‐known isoprenoids. There are many industrial applications, and isoprenoids can also be used in liquid fuels. In eukaryotes and archaea isoprenoids are mainly biosynthesized from a common intermediate known as mevalonic acid in the so‐called mevalonate pathway (MVA). Acetyl‐CoA is converted in sequential steps to 3‐hydroxy‐3‐methyl‐glutaryl‐CoA, which is then converted to mevalonate. Finally, after successive phosphorylations and a decarboxylation, it is formed isopentenyl diphosphate (IPP) and dimethylallyl diphosphate (DMAPP), the fundamental units for isoprenoid biosynthesis. In fact, all kinds of isoprenoids can be formed by the linkage of different subunits. The methylerythritol phosphate pathway (MEP), also known as deoxyxylulose‐5‐phosphate pathway is most typical of eubacterias and cyanobacterias. It starts from pyruvate and glyceraldehyde‐3‐phosphate, which are converted into deoxyxylulose‐5‐phosphate, 2‐C‐methylerythritol‐4‐phosphate and finally, after several reactions, into IPP and DMAPP (Lange *et al*., 2000; Steinbüchel, 2003; Thibodeaux and Liu, 2009; Gupta and Phulara, 2015).

In bacteria, the production of terpenoids was demonstrated last century in *Rhodopseudomonas*,* Rhodospirillum*,* Rhodomicrobium* or *Chlorobium* and production of pristane and phytane in some *Clostridium* species (Han and Calvin, 1969). However, the first example of a photosynthetic microorganism which is able to produce terpenoids directly from CO_2_ was studied in 2010 by Lindberg and colleagues who demonstrated the possibility of producing isoprene in the engineered cyanobacterium *Synechocystis*.

Although many organisms can produce isoprenoids, they usually do not reach amounts high enough to be commercialized (Peralta‐Yahya *et al*., 2012). In the case of photosynthetic systems, many strategies have been attempted to improve the yields, which consisted on: (i) improving photosynthesis efficiency (identifying key bottlenecks in photosynthesis from both carbon fixation and light capture), (ii) tuning MEP pathway to increase terpene precursors (through the identification and optimization of the expression of the enzymes which are key metabolic bottlenecks in the pathway), (iii) introducing an exogenous MVA pathway to minimize the influence of the MEP pathway regulation, (iv) modifying the key enzyme by means of rational design and directed evolution, and (v) expressing transporters in the membrane to facilitate the secretion of end products (Wang *et al*., 2015).

Furthermore, in order to seek for improved productivities, the isoprenoids synthesis pathway has been strategically expressed into *E. coli* and *S. cerevisae* (Martin *et al*., 2003; Wang *et al*., 2011; Gruchattka *et al*., 2013; Yang *et al*., 2016). Given that the MEP pathway is strongly regulated (Chang *et al*., 2013; Banerjee and Sharkey, 2014), the MVA pathway from yeast was expressed in *E. coli* bypassing regulations and consequently producing IPP and DMAPP at high levels. This platform has been tested for the production of various biofuel isoprenoids compounds, such as bisabolene, farnesene, limonene, pinene, sabinene and isopentenol (Beller *et al*., 2015). An alternative strategy is focused on increasing availability of the subunits IPP and DMAPP by overexpressing the native key enzymes in the endogenous MEP pathway in *E. coli* (Kang *et al*., 2005) and in MVA pathway in *S. cerevisiae* (Peralta‐Yahya *et al*., 2011). It is important to take in account that in a theoretical comparative study of both isoprenoid biosynthetic pathways, the MEP was more competent than the MVA pathway, in terms of stoichiometry, energy consumption and oxidation/reduction balance (Steinbüchel, 2003).

Regardless this result, the company Amyris Biotech has engineered an industrial *S. cerevisiae* strain (PE‐2) to produce farnesane (2,6,10‐trimethyldodecane; C_15_H_32_) using the MVA pathway (Ubersax and Platt, 2010). Farnesane was approved by the ASTM as a renewable jet fuel in 2014 and later by the ANP (*Agencia Nacional do Petroleo*) in Brazil for use in blends of up to 10 per cent with conventional jet fuel kerosene. Farnesane is derived from the conversion of plant sugars into farnesene, which is then hydrogenated into farnesane. Although this represents a promising breakthrough, the production costs of farnesane are still too high to sell it as a commodity.

Microbial production of C_12_/C_14_ and C_16_/C_18_ alcohols has also been explored, mainly as precursors of detergents and surfactants, but they can also be used as bio‐jet fuel precursors. The biosynthesis of fatty alcohols employs a de novo fatty acid biosynthesis pathway, and the first improvements were aimed at achieving higher levels of production by modifications in fatty acid metabolism, similar to those attempts described for alkane(s) production (Steen *et al*., 2010; Zheng *et al*., 2012; Liu *et al*., 2014a; Zhou *et al*., 2016). Other improvements consisted of the optimization of enzymes to achieve production of specific alcohols with the appropriate carbon chain length (Zheng *et al*., 2012). Although direct synthesis of alkanes is a better approach for bio‐jet fuel synthesis, some of the approaches used for the production of fatty acid alcohols can be extrapolated into the biosynthesis of alkanes, such as the optimization of enzymes to modulate the carbon chain or the identification of new enzymes with the best properties.

## Perspectives

### Biotechnological perspectives

The general trend for the different attempts to increase alkane biosynthesis leans towards a clear limitation: the low productions have not yet reached those that could be used in industry, as shown in Table 2, which summarizes the production data from the different examples described in this review. Nevertheless, the achieved amount of alka(e)nes is continually improving, according to the recent reports. This, together with the fact that microorganisms produce C_8_ to C_16_ alkanes, which are the target alkanes for aviation industry, supports the promising advantage of using them as biorefineries. Further advantages are that some microorganisms can produce alkanes directly from sunlight and CO_2_ or from wastes, which significantly reduces the carbon footprint impact of their production. As such, intense research efforts should be continued to improve and develop an efficient technology that will be able to provide jet fuels in the future.

Although at this stage of research it is difficult to determine with precision which are the most limiting factors in alkane production, it seems clear that FA biosynthetic and/or degradation pathways are clear targets to overproduce alkanes. In addition, limitations may be caused by poor activity of the enzymatic functions in charge of the alkane biosynthesis (ADO), and/or the side pathways that produce high levels of alcohols instead of alkanes.

Alkane production is derived from intermediaries of fatty acids metabolism therefore overexpression of genes that represents a bottleneck in the fatty acids biosynthetic and/or degradation pathway might enhance the final synthesis of alkanes. A positive example of this is the outcome of the acetyl‐CoA carboxylase (AccABCD) enhancement in *Synechocystis* sp. PCC6803 (see above), this key enzyme in fatty acid biosynthesis showed a clear impact in yield improvements (Cao *et al*., 2014). It has also been suggested that FabH and FabI are inhibited by fatty acyl‐ACPs, and the removal of this inhibition (by overexpression of the genes or modification of the enzymes) could also be key in the enhancement of FAS (Lennen and Pfleger, 2012). In fact, overproduction of FabI, but not FabH seems to have an enhancing role (Cao *et al*., 2016); furthermore deletion of *fadR* also improved alkane production (Choi and Lee, 2013).

In terms of enzymatic enhancement, a global conclusion that can be extracted from most of the engineered pathways is that the decarbonylase ADO enzyme may not be sufficiently robust to produce the required yields. Most of the cases shown above indicate that aldehydes are either transformed to alcohols by other enzymes, or not further metabolized. Hence, finding alternative enzymes with more affinity and activity, or tweaking the amino acids of known enzymes should take top priority in the field. Moreover, the need of improving the electron transfer system for ADO activity has to be considered.

In our view, either finding alternative enzymes or working with characterized ADO, the future of efficient biotechnological platforms passes through a necessary understanding of the metabolic fluxes, and how much of the carbon could be derived towards biofuel synthesis. A prior understanding of metabolic networks is the key before learning how to tweak the background microorganism. In addition, under the perspective of the newest results (Cao *et al*., 2016), it would be interesting to reanalyse how the circulation of intermediates (aldehydes) and side products (alcohols) are driven, since it appears that dynamic balances between the different compounds is more beneficial than drastic re‐channelling of the carbon flows.

An excellent example of how biotechnological optimization can lead to market‐worthy yields of bio‐products is butanol, which is also derived from acetyl‐CoA. Butanol has been successfully synthesized in yeast with profitable yields as high as 18.6 g l^−1^; the strain used has multiple genetic alterations such that it overexpresses heterologous genes to reach 80.3% of the maximum theoretical yield (Lies *et al*., 2012). Although this strain is dedicated to the production of butanol, recent work from Zhou *et al*. (2016) is making inroads towards manipulating the yeast central metabolism and tuning the carbon flow towards production of fatty acids. The high productivity of alkanes in oleaginous yeasts demonstrates the theoretical productivity that could be attained and provides clues for how commercializable yields could be achieved.

Despite efforts in improving current platforms, another important strategy to increase yields is the identification and development of new microbial platforms. Recently, the bacteria *Cupriavidus necator* (also known as *Ralstonia eutropha)* has been used as microbial platform for the production of alkanes with similar results to those reported in the most efficient recombinant *E. coli* described above (Crépin *et al*., 2016). *C. necator*, a chemoautotrophic bacteria that has been used previously to produce chemicals from CO_2_, accumulates PHB at high concentrations (61 g l^−1^, almost 70% of total CDW) when growth conditions for unfavourable. PHB production derived from fatty acid metabolism and using custom‐developed genetic expression tools (Bi *et al*., 2013), significant alkane yields (pentadecane and heptacane) have been achieved; furthermore, a more recent paper (Crépin *et al*., 2016) described a *C. necator* strain that was able to produce a total of 670 mg l^−1^ of hydrocarbons, 435 of which are alkanes. With the aim of achieving similar levels of alkane expression, the ADO and AAR from *S. elongatus*, were overexpressed in a strain lacking the genes for the synthesis of PHB—this ensured that acetyl‐CoA was directly shunted towards the synthesis of hydrocarbons. Alkane synthesis was further favoured through growth in a nitrogen unbalanced media, which limited growth. A layer of decane was also present in the media in order to extract the alkanes produced, which further enhanced yields. Of the recovered product profile there was a high proportion of hexadecanal (235 mg l^−1^), which suggests, again, that the aldehyde decarbonylase ADO is a bottleneck in the process. Crépin and colleagues confirmed that alkane was produced directly from CO_2_, although at low levels (and the scenario that was tested did not include the decane layer). Importantly, *C. necator* is the first example of a recombinant organism that is able to produce alkanes directly from carbon dioxide.

Finding new bacteria from highly specific environmental niches that possess enhanced alkane production capabilities is another strategic focus. Exploration of new niches could be used in the search for better hosts or in the identification of more powerful enzymes that are more active or that have different substrates specificities. For example, rumen microbiota has been recently explored as a source of possible consolidated bioprocesses. Because this environment is rich in cellulolytic microorganisms, it would be expected to harbour microorganisms capable of directly transforming vegetal fibres into biofuels. In addition, an innovative, thermodynamics‐based approach to isolate new alkane‐producing microorganisms has been recently explored by Kohn and Kim (2015). The authors hypothesized that, because many fermentation systems are close to thermodynamic equilibrium, when the systems are perturbed, by, for example changing the concentrations of substrates, the system readjusts in order to restore the equilibrium. According to this principle, they isolated microorganisms able to degrade alkanes in favourable conditions. Once theses microbial platforms were established, the authors altered fermentation conditions by changing the proportions of H_2_ and CO_2_ to make the reverse reactions thermodynamically optimal. Based on this principle the authors found 46 different isolates that were able to produce C_3_ to C_8_ hydrocarbons, including 1‐hexane directly from H_2_ and CO_2_. Of the isolates able to produce alkanes, they identified species of *Actinomyces sp*., *Enterococcus faecium*,* E. hirae*,* E. coli*,* Clostridium glycolicum*,* Proteus sp*. and *Tissirella sp*. Further studies are required to confirm whether these strains are alkane producers and to define their metabolic pathways in order to identify useful enzymatic functions and to glean vital information for the improvement of existing recombinant strains.

While the use of alternative microorganisms, such as oleaginous yeast, for industrial production of alkanes and other jet fuels holds much promise, the advancement of this approach is currently limited by the lack of basic research and knowledge about these new potential bioplatforms. Knowledge gaps must be filled for these microorganisms in terms of genetics, basic metabolism and the pathways engaged in oil production, as well as in the development of the necessary tools to tune the overexpression of specific functions (Sheng and Feng, 2015). We believe that the present demand for bio‐fuels may be able to be quenched only when these new high‐potential bacteria and yeast are properly and fully characterized.

### Bio‐jet fuel global perspectives

The current drivers of bio‐jet fuel research include the global push to reduce GHG emissions; and the diversification of fuel suppliers to normalize prices (Gegg *et al*., 2014). In terms of fuel supplier diversification, bio‐jet fuels provide two main advantages: reducing oil imports and price stabilization. By using renewable instead of fossil fuels, regional economies would experience significant gains due to the engagement of new agricultural and/or industrial processes. Each region, however, has different natural conditions, resources and potentials, and therefore, the identification of proper production processes and appropriate technology for aviation biofuels is essential. For example, Europe has a relatively small surface area with limited natural resources; however, there are around 500 million inhabitants and each one produces around 500 kg of garbage per year. Reutilization of waste to produce biofuels represents a game‐changing approach that will greatly enhance and stabilize Europe's economy. Once these renewable fuels are market‐ready, volatility of fossil fuels will decrease, allowing the aviation industry to plan costs and better use resources.

Legislation could be another important driving force to implement the utilization of bio‐jet fuel, however, there is a perceived lack of national and international political coordination to support aviation biofuels. US airlines are not subject to significant environmental national legislation (Gegg *et al*., 2014), whereas in Europe, Emission Unit Allowances (EUA), which are assigned to the airline sector by the European Emissions Trading System (ETS) account for 97% of their historical CO_2_ emissions (average 2004–2006) and will be reduce to 95% during the 2013–2020 period. However, actual emissions are lower than historical records and airlines are allowed to accumulate previous years EUAs. As a consequence, there is minimal pressure on airlines to comply with the ETS, at least in Europe, which ultimately promotes weaker legislation pressure regarding CO_2_ emissions. Agreement between the different international agencies to establish a common protocol and enforce it with legislation might achieve the desired emissions limits.

Although certain types of bio‐jet fuels have become fully certified for commercial use, aviation biofuels are still far from commercialization. It is clear that current technologies for bio‐jet fuel synthesis are limited by the cost of the process and the availability of suitable feedstocks. The synthesis of bio‐jet fuels from wastes (industrial or agricultural) rather than from oily crops or algae will provide real advantages, such as energy savings, lower costs and avoidance of setbacks such as catalyst poisoning (which occurs in the FT process). This approach, while still yet to be realized, represents a highly attractive approach in terms of potential economic benefits and competitiveness versus existing fossil fuel‐based fuels that are currently in the market.

## Concluding remarks

It is evident that the production of biofuels from crops, which takes land away from food production is not viable in the long‐term. Thus, the identification of alternative renewable carbon sources is essential for making bio‐jet fuels a reality. The improvement of 2G bioethanol technology has paved the way for the utilization of waste (from agriculture or municipalities) for the production of other value‐added products. Production of sugars from these renewable waste sources is now a fact, and the efficient and economically viable utilization of these sugars for hydrocarbon production is the next challenge that biotechnologists must overcome. With the appropriate political and societal climate, the development of better microbial platforms for the production of alkanes will translate into improved production processes.

Despite all the considerations and current inefficiencies, there is an unquestionable interest in continued research into alkane biosynthesis—this is being driven by economic and social factors, but also because of recent biotechnological advances that can be applied within the field. There is no doubt that research in the synthesis of renewable jet fuels is a hot topic in biotechnology—a reality that is reflected in the increasing number of publications and patents published over the last 20 years. As an example, many relevant papers cited in this review have been published in the last few years. Although, as we have pointed out, there exist serious limitations in strains currently in use, the most recent papers cited here have made particularly important advances at identifying and describing new strains and species, which suggest that there is still much to explore. We believe that unstoppable advances in biotechnology and synthetic biology, together with the enormous demand for bio‐fuels will foster a bright future for bio‐jet fuel production.

## Conflict of Interest

None declared.
